# Preputioplasty as a surgical alternative in treatment of phimosis

**DOI:** 10.1038/s41443-021-00505-9

**Published:** 2021-12-01

**Authors:** Daniar Osmonov, Claudius Hamann, Ahmed Eraky, Almut Kalz, Diethild Melchior, Robert Bergholz, Javier Romero-Otero

**Affiliations:** 1 Department of Urology and Pediatric Urology, University hospital Schleswig-Holstein, Campus Kiel, Kiel, Germany; 2 Department of Pediatric Surgery, University hospital Schleswig-Holstein, Campus Kiel, Kiel, Germany; 3 Urology Department, Hospital Universitario 12 Octubre, Instituto de Investigación Sanitaria Hospital 12 de Octubre (imas12), Madrid, Spain

**Keywords:** Urogenital diseases, Reproductive disorders

## Abstract

Preputioplasty denotes various surgical techniques directed at resolving phimosis without the need for radical or partial circumcision. This narrative review summarizes the best-known surgical techniques of preputioplasty. A MEDLINE and EMBASE-based literature search of original manuscripts and case reports published in English has been carried out using the following key words: “circumcision”, “partial circumcision”, “phimosis”, “paraphimosis”, and “preputioplasty”. Six different procedures are explored in more detail and illustrated. The complication rates of all surgical procedures presented here are reported to be low. In cases of medical (rather than cultural and religious) indications, foreskin-preserving procedures present useful alternatives to circumcision in the routine clinical practice of urologists and pediatric surgeons.

## Introduction

Surgical treatment of phimosis is a very common procedure worldwide. Radical or partial foreskin removal is one of the oldest surgical procedures in the history of mankind. The foreskin was called “prepuce” in Roman time and means as it protrudes before (pre) the tip of penis (putos) [1]. The inventor and first person to perform a circumcision is unknown. The actual indication for circumcision was not a medical condition but was based mostly on religious and cultural beliefs [2, 3].

Testify to the known historical sources, circumcision was first performed in old Egypt probably inspired by the mythology of Osiris. From those old times till now circumcision is an important part of the Jewish and Muslim cultures [3]. Based on the historical documents of the 19th century, circumcision was known to be performed as “ultima ratio” for masturbation, seizures, epilepsy, and paraplegia. Only in the modern time, beginning form the middle of the 20th century, circumcision has become a medical indication as a surgical option in the treatment of phimosis [3]. Circumcision is the most-performed surgical procedure in modern medicine [4]. The main indications are: non-retractable foreskin due to phimosis or paraphimosis, still as a part of cultural and religious beliefs, and finally as a prevention of penile cancer, sexually transmitted diseases [4].

There are a number of non-surgical alternatives to circumcision that have been described in the literature, such as a retraction therapy, variations of the steroid applications, and finally systemic antibiotics are recommended in case of balanoposthitis [5–10].

All the latter treatments have the goal to retract the foreskin and do not aim at the removal of the entire foreskin. Alternative surgical treatments include different types of preputioplasty. The term preputioplasty denotes various surgical techniques directed at resolving phimosis without radical or partial circumcision. This narrative review summarizes the best-known surgical techniques of preputioplasty, such as triple incision plasty, preputial plasty, ventral “V”-plasty (“VVP”), “Y”-“V” plasty, trident plasty, and “Z”-plasty, exploring the success rates and complications of the known procedures.

## Materials and methods

### Literature search and study eligibility

A MEDLINE and EMBASE-based literature search of original manuscripts and case reports published in English has been carried out using the following key words: “circumcision”, “partial circumcision”, “phimosis”, “paraphimosis”, and “preputioplasty”.

### Data extraction

All subsequent articles including case reports which describe a surgical technique were cross-referenced to ensure capturing of all relevant papers. In general, 16 articles regarding to the topic of the current review were identified. Only 9 original research articles reflecting current evidence without considering the time of publication were included. Case reports or original articles with low number of patients were excluded from the evaluation. The available articles were catalogued in a table which included the name of the journal, the number of patients, the type of reconstruction as well as the success rate. In this review, we will demonstrate and discuss the different surgical procedures.

### Surgical procedures

1. Triple incision plasty as first described by Nils Wåhlin (1991) [11]. The foreskin is gently retracted until it is too narrow to pull any further (Fig. 1). Three longitudinal incisions are made as demonstrated (Fig. 1). It is recommended to sever all transverse structures until the foreskin can be moved (Fig. 1). Each incision is closed by somewhat oblique locking stiches, thereby rotating around the foreskin and the suture lines so that they lie parallel to each other obliquely (Fig. 1).

**Fig. 1:**
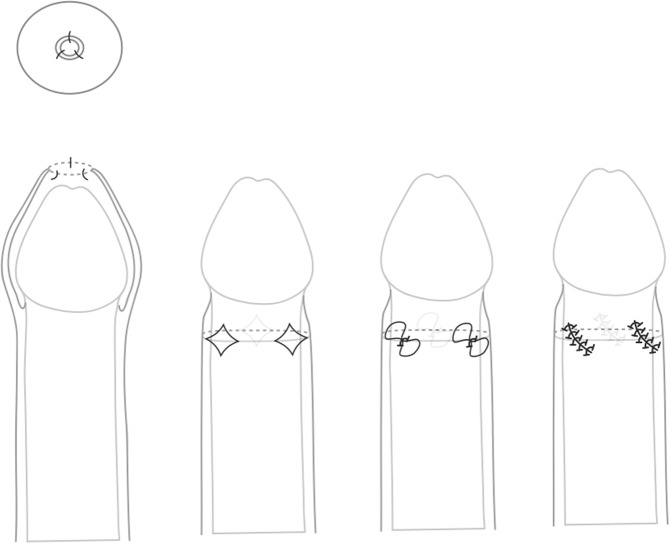
Triple incision plasty (the figure explores the three longitudinal incisions of the foreskin, retracted and movable skin, the place of made incisions, and suture lines).
  Postoperatively, no dressing is applied. There are no specific recommendations for the described Wåhlin procedure, except that the patient should avoid manipulating the foreskin during the first week to achieve proper wound healing [11].
2. Preputial plasty as described by Cuckow et al. (1994) [12]. The foreskin is mobilized by severing the glandular adhesions and retracted (Fig. 2). The constricted tissue is incised longitudinally, alongside the dorsum of the penis. The underlying tissue is spread with artery forceps to expose Buck’s fascia, and the incision is closed transversally using absorbable sutures [12] (Fig. 2).

**Fig. 2:**
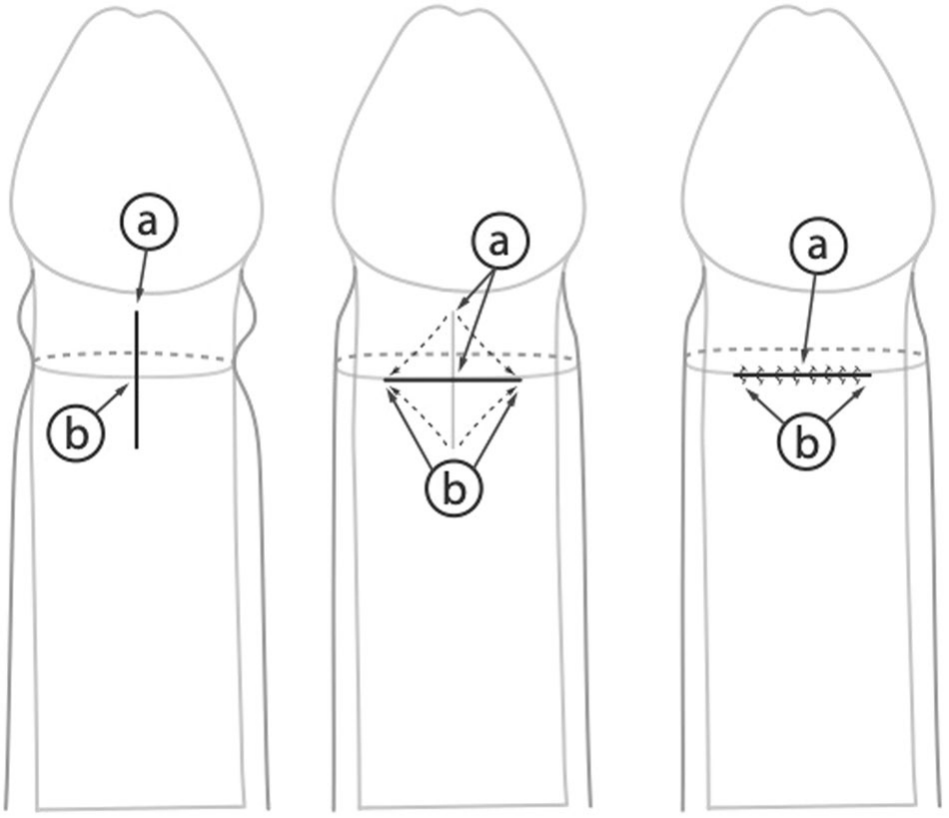
Limited dorsal slit (mobilized the foreskin by performing longitudinal incision on the penis dosum, deep incision to expose Buck’s fascia, and transversally closed incision).
  Apart from lidocaine gel applied to the glans and suture line, no other local anesthetic is used. Parents are advised to mobilize the foreskin regularly once the initial discomfort has subsided [12].
3. Ventral V-plasty (VVP) as described by Alexander et al. (2009) [13]. This procedure was proposed as a surgical treatment option for congenital megaprepuce. The VVP technique allows for preservation of the full length of shaft skin [13] (Fig. 3). To preserve this skin, the circumferential incision on the shaft is performed at a level that will ensure sufficient skin length and disregards the constriction tissue. This is then incised in the midline, ventrally as shown in the illustration (Fig. 3). This incision must be of sufficient length to completely divide the area of stenosis. By doing so, a V-shaped defect of variable width and length is created [13] (Fig. 3). Then a circumferential incision is performed on the subcoronar collar at a level that approximates a standard circumcision. This incision is modified ventrally to preserve a V-shaped flap with the exact dimensions of the defect in the proximal ventral shaft skin [13] (Fig. 3). The V-plasty is built by interposing the subcoronar V of skin into the corresponding V-shaped defect in the shaft skin. Traction/apposition sutures are placed into the angles of the V to aid skin closure as illustrated [13] (Fig. 3).

**Fig. 3:**
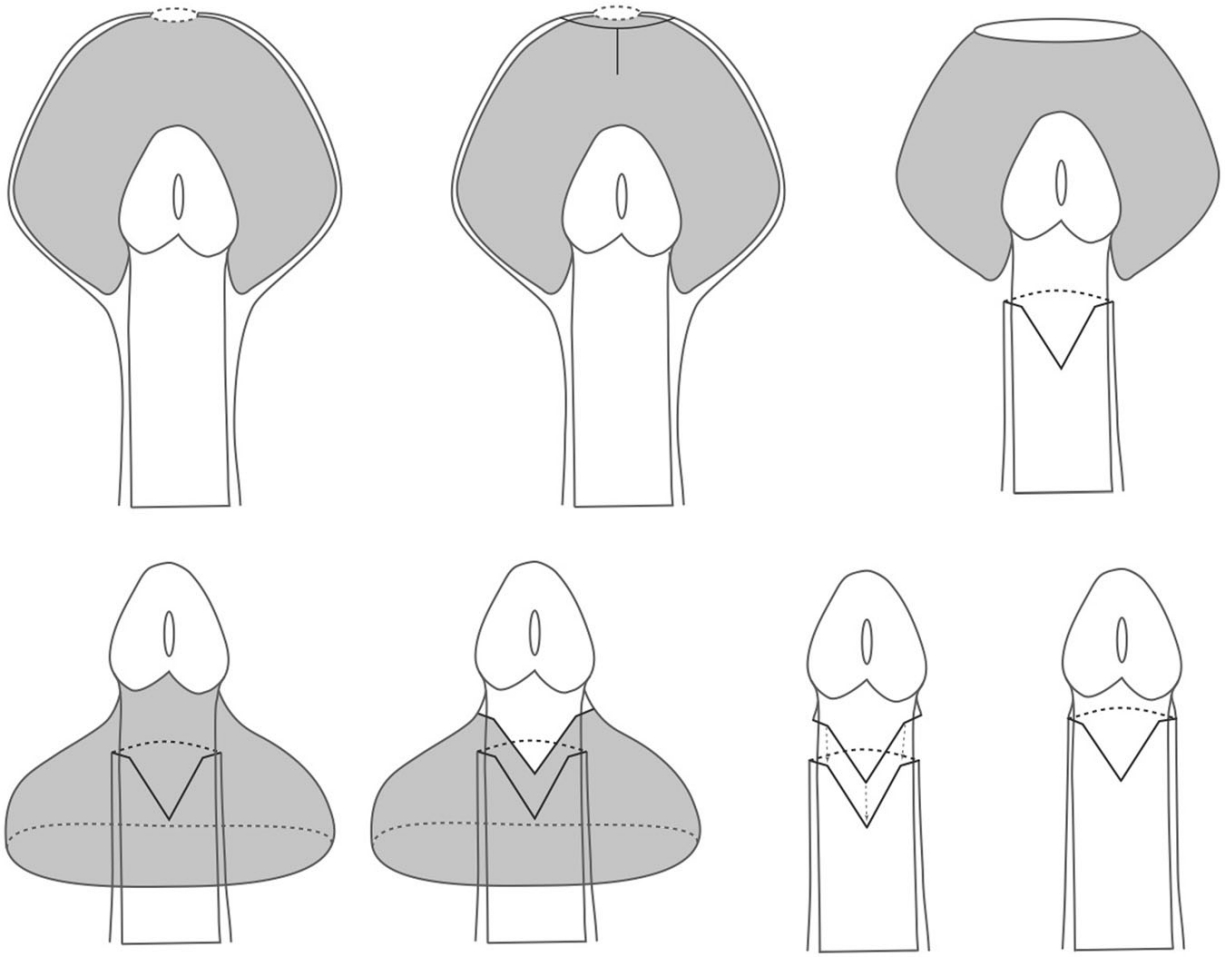
Ventral V-plasty (VVP) for treatment of congenital megaprepuce (circumferential incision of the graft is performed as shown; then a ventral midline incision is performed; a V-shaped defect of variable width and length is created; circumferential subcoronal incision is performed; V-plasty is built by interposing the subcoronal V into the V-shaped defect).
4. Y-V plasty as described by Nieuwenhuijs et al. (2006) [14]. This procedure starts with an inverted “V” with 1 cm “legs” at the narrowest part of the external foreskin, which are then extended to form a “Y” on the inner part of the prepuce [14] (Fig. 4). The tunica dartos layer is severed and the wound is closed as a “V” with six–eight polyglycolic acid sutures (6.0). No dressing is applied. Parents are advised to retract the prepuce daily starting on day 3 [14] (Fig. 4).

**Fig. 4:**
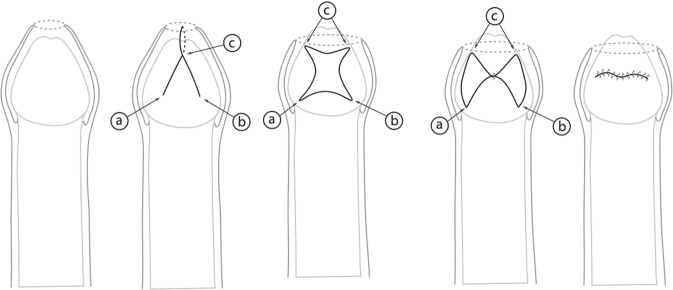
Y-V plasty (characterised by the transformation of the inverted “V” incision to the “Y” on the inner part of the Prepuce.
5. Trident plasty described by Pedersini et al. (2017) [15]. A linear mark is drawn as a transversal line on the proximal side of the prepuce, 2 mm distal to the stenotic ring. The length of this line is approximately one-quarter of the circumference. Three small longitudinal lines were drawn on the distal side of the prepuce [15] (Fig. 5). An inverted “V”, with the apex extended from the perpendicular line, made at the midpoint of the transversal line, and keeping an angle of 60°, is drawn in the proximal prepuce (Fig. 5). It is mandatory for the edges of all flaps to be of the same length. The mucocutaneous flaps of the prepuce are incised, dissected, and then sutured with interrupted polyfilament 6/0 stitches, thus transforming “Y” to “V “[15] (Fig. 5). Patients are discharged on the day of surgery. The follow-up assessments were carried out at 1 and 2 weeks, as well as 1, 6, and 12 months postsurgically [15] (Fig. 5).

**Fig. 5:**
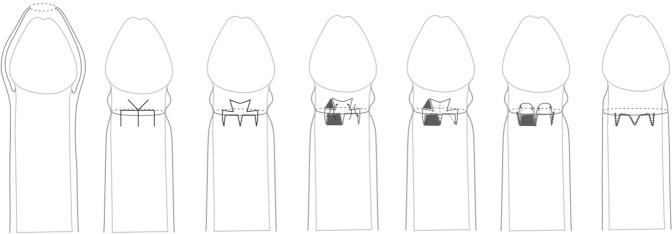
Trident preputial plasty (an inverted “V” is made at the midpoint, full-thickness flaps of the prepuse are incised and dissected, and transformation of “Y” to “V” is performed).
6. Z-plasty described by Emmett (1982) [16]. The principle of this procedure is based on the Heineke-Mikulicz principle of lateral incisions made longitudinally and closed transversally [16, 17] (Fig. 6). The scarred phimotic ring is excised, resulting in a circular incision [16, 17] (Fig. 6). Z-plasties are performed at 3 and 9 o′clock positions. Two flaps of equal dimension are created [16, 17] (Fig. 6). The flaps are then mobilized, rotated, and transposed to the contralateral apex, and finally sutured in place with a 6/0 chromic suture (Fig. 6). A compressive dressing with gauze and tegaderm is applied. All patients are discharged on the day of surgery.

**Fig. 6:**
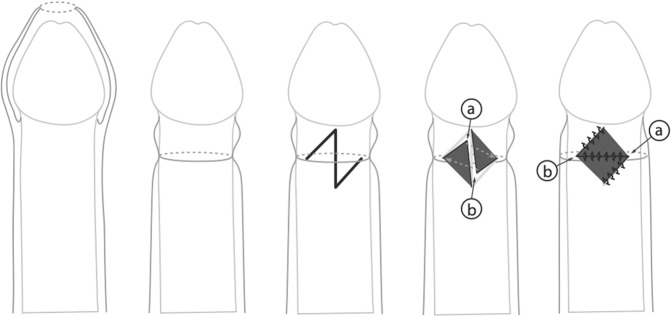
Z-plasty (based on the lateral incision made longitudinally and closed transversally).

## Discussion

The treatment options of phimosis are not limited to radical or partial circumcision [5–10], which—while being a quick and straightforward solution—should not be the only one. Moreover, current clinical recommendations from pediatric surgeons recommend preputioplasty as the method of choice with the goal to achieve retractibility of the foreskin [1]. Our aim was neither to favorize one over the other treatment options, nor to underestimate the role of circumcision as a radical surgical option, but to present the best-described options from which we can choose in daily clinical routine. Without doubt, the topical treatment of phimosis is a first-line treatment in pediatric practice. Such medical approaches include a topical corticoid cream (betamethasone 0.05–0.1%) applied twice a day over a period of 20–30 days (LE: 1; GR: A) [5–10]. This treatment has no side effects, and, which is very important to mention, it does not increase the mean bloodspot cortisol levels (LE: 1) [10].

The treatment of phimosis is multimodal and should be adapted to the clinical and individual situation, considering the presence of local infections, cultural and religious aspects as well as the patient’s respective parents’ preferences. Medical consultation must explore all known treatment options in the treatment of phimosis and should be clearly documented [1, 2].

Different methods of preputioplasty are useful and can be recommended and performed in nearly all cases of phimosis [11, 18] (Table 1). Preputioplasty procedures can be categorized into procedures with either a single incision and subsequent suture, or multiple incisions (two or more) around the circumference, based on geometrical patterns such as “V”/”Y”, “Z”, or complicated sliding plasty [11, 18] (Table 1). Procedures with multiple incisions have been invented to apportion the widening of the constriction more consistently to the whole circumference and thus to achieve better functional and cosmetic outcomes [11]. Despite numerous plasties previously described, not all of them seem to have found their way into general use, probably due to technical or cosmetic drawbacks.

**Table 1:** Preputioplasty studies.

Author	Year	Study design	Follow-up	Complication	*N*	Type of reconstruction	Success rate
Benson et al., Journal of Pediatric Urology [17]	2018	Retrospective	6 Months	Scarring in 1/28 patients (pat.)	28	Z-plasty	98%
Pedersini et al., Journal of Pediatric Urology [15]	2017	Prospective	12 Months	Scarring in 1/41 pat.	41	“Trident” preputial plasty	97.6%
Stewart et al., Urology [19]	2012	Retrospective	26 Months	a. Standard preputioplasty: scarring in 5/22 pat. b. Z-Plasty scarring in 1/12 pat.	22 vs. 12	Standard vs. Z-plasty	82%
Monarca et al., Gironale di Chirurgia [20]	2013	Retrospective	6 Months	No pathological scarring	52	Simple running suture	92%
Nieuwenhuijs et al., Journal of Pediatric Urology [14]	2007	Retrospective	6 Months	Scarring in 2/47 pat	47 vs. 18	Y-V plasty vs. transverse closure of longitudinal incisions of the narrow preputial ring.	95.7% vs. 89%
Cuckow et al., Journal of Pediatric Surgery [12]	1994	Retrospective	N/A	Scarring in 2/50 pat.	50 vs. 50	Circumcision vs. limited dorsal slit	N/A
Alexander et al., Journal of Pediatric Surgery [13]	2010	Retrospective	6 Months	None	10	Ventral V-plasty	N/A
Erdenetsetseg et al., Journal of Urology [21]	2003	Retrospective	12 Months	Skin fistula in 3/51 pat.	51	N/A	70.6%
Nils Wåhlin, Scandinavian Journal of Urology and Nephrology [11]	1992	Retrospective	6 Months	N/A	63	Triple incision plasty	N/A

Triple incision plasty is one of the very common variations of the preputioplasty and was first published by Nils Wåhlin, a Swedish pediatric surgeon [11] (Fig. 1). In the original publication, 63 patients between 2 and 27 years old were evaluated. Major surgical complications according to the current Clavien-Dindo score were not reported [11] (Fig. 1). There were also no Clavien-Dindo 2 complications that needed revision. One patient had a slightly prolonged bleeding for 2 days, 2 patients showed swellings and one incurred a superficial infection, the total of all complications was 6/63 = 9.5% with a follow-up of at least 1 year [11] (Fig. 1).

The next procedure by Cuckow et al. describes a simple preputial plasty [12]. In effect, it is a simple dorsal slit. The procedure is easy to perform and widely used in both adult and pediatric urology. In all, 50 patients were evaluated retrospectively in the initial paper [12]. The authors compare the outcome of the simple preputial plasty to that of classic circumcision. Evaluation regarding the operative morbidity and patient satisfaction was obtained by sending a questionnaire to all patients’ respective parents. No complications of Clavien-Dindo 2 or higher were reported in the group of preputial plasty, whereas 6% (*N* = 3) of the patients in the circumcision group were reported to have required surgical revision due to bleeding problems [12] (Table 1). The respective distribution of complications in the preputioplasty vs. the circumcision group was as follows: infections (10% vs. 12%), huge edema (2% vs. 0%), recurrent adhesion (2% in both), a non-retractile foreskin (4% vs. 0%), and poor cosmetics (2% vs. 6%) [12].

In cases of a congenital megaprepuce with a concomitant buried penis, the VVP was described as a method of choice by Alexander et al. [13] (Table 1). In the initial evaluation, he described the surgical outcome in 10 children. Parental satisfaction was high in 10/10 children. One child required a secondary minor cosmetic procedure. No complications were reported.

The study on Y-V plasty was carried out in 65 cases [14] (Table 1). The presented Y-V technique was compared to the transversally closed longitudinal incisions on the narrow part of the prepuce [14]. Revision surgery in the Y-V group was 4.3% and 11% in the control group. No major complications were reported in either group. The cosmetic results were excellent in all Y-V cases performed [14].

One of the oldest variants of preputioplasty is Z-plasty [16]. There are but few of studies describing the efficacy of Z-plasty. In a recent study, a cohort of 28 patients was described with a follow-up of 24 months [17]. All patients showed satisfactory wound healing without infections, hematoma, or flap necrosis. All patients had previously failed to respond to the topical treatment with betamethasone. During follow-up, the prepuce was fully retractable in all patients [17].

The trident plasty, at last, presents a combination of the afore-described Y-V plasty and Z-plasty without diminution of the surgical outcomes during the assessed follow-up [15].

Comparison of the outcome of different surgical options confirms that the single plasties, which are essentially equivalent to a dorsal slit and easy to perform, tend to give a cosmetically unsatisfactory result, with a visible cleft or deformity. Radical circumcision, by contrast, carries a higher risk of complications, among them is, for example, fibrotic healing. Therefore, the surgical options that preserve the foreskin should be given priority in the treatment of non-complicated phimosis.

### Limitations

In general, evidence is poor and based on retrospective, single-center studies with a limited number of patients as well as on case reports. Moreover, the studies are limited from reporting success rats as well as complication rates based on non-standardized criteria. Nevertheless, current article explores all known surgical techniques of preputioplasty and may have a practical guidance in the daily clinical routine.

## Conclusion

Various surgical options are available for preputioplasty. The complication rates of all surgical procedures presented here are reported to be low. In cases of medical (rather than cultural and religious) indications, foreskin-preserving procedures present useful alternatives to circumcision in the routine clinical practice of urologists and pediatric surgeons.
